# Personal

**Published:** 1916-09

**Authors:** 


### PERSONAL

---

Announcement of removal, travel, and other matters of interest are requested. Please report errors in the listing of any physician in the State and other directories, that we may co-operate with the State Society in securing a correct list.

---

Dr. Frank C. Kleckner of Buffalo announces the removal of his office to 2254 Main Street. Practice restricted to diseases of the nose, throat, eye and ear.

---

Dr. J. Walter Fitz Gerald of E. Utica Street, Buffalo, spent July at Georgian Bay.

---

Dr. George Edward Fell of Buffalo has been seriously ill but has recovered sufficiently so that he would be glad to see his friends at his new home, 209 Porter Avenue.

Dr. J. H. Carstens of Detroit has moved his office to 1447 David Whitney Building.

---

Dr. A. E. Woehnert of Buffalo returned from a motor trip to the east, about the last of July.

---

Dr. Burt C. Johnson of Buffalo returned from a two months' trip to Alaska, August 6.

---

Dr. Henry Dowd of Buffalo, moved to 9 N. Pearl Street, in May.

---

Dr. Arnold H. May of Buffalo returned September 1 from New York, where he spent most of August.

---

Dr. James Wright Putnam of Buffalo spent August in Maine.

---

Dr. Richard J. Gould of Buffalo has moved to 1355 Main Street.

---

Dr. A. E. Clarke of Buffalo has moved to 2300 Main Street.

---

Dr. Edward Torrey of Olean has been appointed Superintendent of the Cattaraugus Tuberculosis Hospital which will occupy a new brick building on the mountain near Rock City.

---

Dr. John W. Le Seur of Batavia, former State Medical Inspector, was appointed August 14, to take charge of the poliomyelitis epidemic in Erie, Genesee, Monroe, Niagara and Orleans Counties.

---

Dr. Edward Clark of Buffalo has been given charge of the poliomyelitis epidemic in Weschester Co.

---

Dr. Julius Richter of Buffalo has been appointed first lieutenant in the Army Medical Reserve Corps.

---

Dr. and Mrs. F. A. Hayes of Landon Street spent the latter part of July and the forepart of August motoring in the Adirondack and Catskill Mountains.

---

Dr. John F. Fairbairn of Buffalo returned, the middle of August, from a month's trip to the Canadian northwest.

---

Dr. Benjamin H. Grove of Buffalo returned early in August from a trip to Atlantic City.

Dr. Homer J. Grant of Buffalo visited the Massachusetts coast in August.

---

Dr. Edith F. Ryan of Batavia, who has recently completed a year's interne service in the Philadelphia Woman's Hospital, has been appointed physician to the Child Welfare Station of Batavia.

---

Dr. Edmund E. Blaauw of Buffalo, returned August 1 from a motor trip to Maine, via the Adirondacks and White Mountains.

---

Dr. Cressy L. Wilbur, formerly chief statistician for the U. S. Census and recently Director of Vital Statistics for the N. Y. State Health Dept. while suffering from melancholia, cut his throat with a razor, July 30 but not fatally.

---

Dr. John R. Simson of Kenmore, had his auto wrecked in a collision, August 19.

---

The automobile of Dr. Cyrus S. Siegfried of Buffalo was stolen in August but was recovered. The report of the theft of the auto of Dr. J. J. Finerty of Buffalo, was incorrect.

---

Dr. John H. Evans of Buffalo announces the removal of his office to 519 Franklin Street. Practice confined to anaesthesia.

---

Dr. Brainerd Hunt Whitbeck, P. & S. 1903, of 40 E. 41, N. Y., is to move to Rochester and occupy the home of his father, Dr. John F. W. Whitbeck recently deceased. Dr. B. H. Whitbeck has devoted his attention to orthopaedic surgery.

---

Dr. Chas. W. Hennington, who has been spending the summer at Pt. Abino, Ont., has returned to Rochester.

